# The omnivorous predator *Macrolophus pygmaeus*, a good candidate for the control of both greenhouse whitefly and poinsettia thrips on gerbera plants

**DOI:** 10.1111/1744-7917.12655

**Published:** 2019-01-20

**Authors:** Ada Leman, Barbara L. Ingegno, Luciana Tavella, Arne Janssen, Gerben J. Messelink

**Affiliations:** ^1^ Wageningen University & Research Business Unit Greenhouse Horticulture Bleiswijk the Netherlands; ^2^ Dipartimento di Scienze Agrarie, Forestali e Alimentari (DISAFA), ULF Entomologia Generale e Applicata University of Torino Grugliasco (TO) Italy; ^3^ IBED, Department of Evolutionary and Population Biology University of Amsterdam Amsterdam the Netherlands

**Keywords:** apparent competition, biological control, *Echinothrips americanus*, pest interactions, prey preference, *Trialeurodes vaporariorum*

## Abstract

The poinsettia thrips *Echinothrips americanus* Morgan is a relatively new pest that has spread rapidly worldwide and causes serious damage in both vegetable and ornamental plants. In this study, we investigated if and how effective this pest can be controlled in gerbera by the omnivorous predator *Macrolophus pygmaeus* (Rambur). Because herbivores on plants can interact through a shared predator, we also investigated how poinsettia thrips control is affected by the presence of the greenhouse whitefly *Trialeurodes vaporariorum* (Westwood), a pest that commonly coexists with *E. americanus* in gerbera. In laboratory studies, we found that the predator *M. pygmaeus* fed on both pests when offered together. Olfactometer tests showed a clear preference of the predators for plants infested by whiteflies but not by thrips. In a greenhouse experiment, densities of both pests on single gerbera plants were reduced to very low levels by the predator, either with both pests present together or alone. Hence, predator‐mediated effects between whiteflies and thrips played only a minor role. The plant feeding of the shared predator probably reduced the dependence of predator survival and reproduction on the densities of the two pests, thereby weakening potential predator‐mediated effects. Thus, *M. pygmaeus* is a good candidate for biological control of both pests in gerbera. However, further research is needed to investigate pest control at larger scales, when the pests can occur on different plants.

## Introduction

Generalist predators have become important biological control agents in greenhouse crops both because of their ability to control multiple pest species and their capacity to persist in crops prior to pest invasions by feeding on alternative food sources (van Lenteren, 2012; Messelink *et al*., 2014). Such preventive releases or “crop inoculations” with generalist predators have proven to be very effective for controlling several pest species (Ramakers & van Lieburg, 1982; van den Meiracker & Ramakers, 1991; Calvo *et al*., 2012; Messelink & Janssen, 2014). An important group of generalist predators that are used in greenhouse crops are the predatory bugs of the family Miridae of the tribe Dicyphini (belonging to the *Nesidiocoris*, *Dicyphus*, and *Macrolophus* genera) (Castañé *et al*., 2004; Ingegno *et al*., 2009; van Lenteren, 2012; Messelink *et al*., 2015). They attack a wide range of prey, including important greenhouse pest species such as whiteflies, aphids, thrips, and lepidopteran pests (Riudavets & Castañé, 1998; Montserrat *et al*., 2000; Urbaneja *et al*., 2009; Ingegno *et al*., 2013; Messelink *et al*., 2015; Ingegno *et al*., 2017). Moreover, because they are true omnivores that feed on both plant and prey, they can even survive in some crops without prey (Coll & Guershon, 2002). Another benefit of these omnivores is their potential role in controlling invasive pest species, which was shown when the South American moth *Tuta absoluta* (Meyrick) appeared in Europe (Zappalà *et al*., 2013).

In this study, we evaluated whether the omnivorous predator *Macrolophus pygmaeus* (Rambur) (Hemiptera: Miridae) can be used to control the poinsettia thrips *Echinothrips americanus* Morgan (Thysanoptera: Thripidae), which is a relatively new pest (since 1995 in Europe and 2010 in China) that has spread rapidly worldwide and causes serious damage in both vegetable and ornamental plants (Vierbergen *et al*., 2006; Li *et al*., 2014). In Northern Europe, it is one of the emerging pests in ornamental crops such as roses and cut gerbera flowers. Controlling this thrips species by predatory mites has not been very effective so far (Opit *et al*., 1997; Hoogerbrugge *et al*., 2014; Ghasemzadeh *et al*., 2017). Hence, there is a need for additional, better biological control agents and mirid predators may be suitable candidates, because they proved to be effective against other thrips species (Riudavets & Castañé, 1998; Messelink & Janssen, 2014; Bouagga *et al*., 2018). In greenhouse gerbera, *E. americanus* commonly coexists with the greenhouse whitefly *Trialeurodes vaporariorum* (Westwood) (Hemiptera: Aleyrodidae), which makes it essential to evaluate how the control of *E. americanus* is affected by the presence of whiteflies and vice versa, because indirect interactions may occur when pests share a predator (Messelink *et al*., 2008). Moreover, pest performance may be affected by plant feeding by this omnivore (Pappas *et al*., 2015; Zhang *et al*., 2018).

Based on the predator's prey preference and the relative abundance of pests, predators may temporarily concentrate their attacks on one pest, which allows the other pest to escape from predation (Murdoch, 1969; Abrams & Matsuda, 1996; Jaworski *et al*., 2013). Such dynamics may even disrupt biological control in the short term, as shown for other generalist predators (Madsen *et al*., 2004; Koss & Snyder, 2005; Symondson *et al*., 2006; van Maanen *et al*., 2012b). In the long term, the diversity of pest species is usually beneficial for pest control (Messelink *et al*., 2008; Chailleux *et al*., 2014; Munoz‐Cardenas *et al*., 2017), because it will result in higher densities of the shared predator and lower densities of the pests that share this predator [apparent competition (Holt, 1977)]. Because of these potential predator‐mediated interactions, we assessed predation rates of *M. pygmaeus* on both pest species when offered together in the laboratory and studied the preference of the predators for plants with either of the two species in olfactometer tests. Furthermore, we evaluated the control of single and combined populations of both pests on gerbera plants. To detect the possible additional role of plant‐mediated pest interactions, we also included treatments with single and combined pests on plants without predators.

## Materials and methods

### Plants and insects

All plants used for maintaining insect colonies and for experiments were cultivated in heated experimental greenhouse compartments of Wageningen University & Research at 24 ± 1 °C and 60% ± 5% relative humidity (RH). Colonies of *M. pygmaeus* were established starting from individuals purchased from Koppert Biological Systems (Berkel en Rodenrijs, the Netherlands) and reared on flat pods of *Phaseolus vulgaris* L. (Fabaceae) inside transparent plastic cylinder boxes (Ø 250 mm × 270 mm) (JET 107 PM, Jokey Plastik GmbH, Sohland, Germany). Ventilation was possible through a hole in the lid covered with insect gauze (mesh size 80 μm). Individuals were fed with sterilized eggs of *Ephestia kuehniella* Zeller (Lepidoptera: Pyralidae), purchased from Koppert Biological Systems (Berkel en Rodenrijs, the Netherlands). The predators were reared in a climate chamber set at 25 °C, 70% RH, with a 16 : 8 h (L : D) photoperiod. Colonies of *T. vaporariorum*, originally collected from tomato plants in a greenhouse in the province of South Holland, were reared on gerbera plants in insect cages (600 × 600 × 900 mm) (Type 80.304, Vermandel BV, Hulst, the Netherlands). Colonies of *E. americanus* were started with individuals collected from ornamentals in greenhouses in the South Holland region and reared on gerbera and pepper plants enclosed in insect cages in the greenhouse. Mass rearings of whiteflies and thrips were maintained in a heated greenhouse (24 ± 1 °C; 60% ± 5% RH).

### Predation rates

The daily consumption of whiteflies and thrips by *M. pygmaeus* females was assessed in the laboratory of Wageningen University & Research to study whether the predator ate both prey when both are abundantly present. One‐week‐old *M. pygmaeus* females were individually starved in a glass vial with some water‐soaked cotton wool for 16 h to ensure that they were motivated to feed. The experiment was conducted in 280‐mL plastic boxes (Ø 80 × 50 mm) (Paardekooper Verpakkingen B.V., Oud‐Beijerland, the Netherlands) with a gerbera leaf disc (cv Kimsey, Terra Nigra B.V., Kudelstaart, the Netherlands) on water‐soaked cotton wool. Ventilation was possible through a hole in the lid, covered with insect gauze (mesh size 80 μm). Each box was provided with 50 pupae of *T. vaporariorum* and 50 adults of *E. americanus*, which were gently transferred with a tiny brush onto the leaf disc. Subsequently, each box was provided with one starved mirid female. Predation rates were assessed for 10 female predators. The boxes were placed in a climate chamber under 16 h of artificial illumination per day at 25 °C and 70% RH. Predation of thrips and whiteflies by the mirid, recognized by the presence of bodies that were sucked empty, was assessed after 24 h under a stereomicroscope after removal of the predators. Predation of whiteflies by thrips, which has been observed for western flower thrips *Frankliniella occidentalis* (Pergande) (Thysanoptera: Thripidae) (van Maanen *et al*., 2012a), was never observed for *E. americanus*.

Consumption of whitefly pupae and thrips adults was analyzed by calculating Manly's index (Manly, 1974):

$$\beta_{1}=\frac{\log\left(e_{1}/A_{1}\right)}{\log(e_{2}/A_{2})+\log(e_{1}/A_{1})},$$

where *β*_1_ is the consumption of prey type 1, *e*_1_ and *e*_2_ are the numbers of prey type 1 and 2 remaining after experimentation, *A*_1_ and *A*_2_ are the initial number of prey type 1 and 2. The value of *β* will fall between 0 and 1, with *β* = 0.5 representing equal predation of both prey. One‐sample *t*‐tests were used to test the significance of Manly's index under the expectation that the mean of Manly's index was equal to 0.5.

### Response to plant volatiles

Olfactometer tests were conducted in the laboratory of DISAFA, University of Torino. In the bioassays, active *M. pygmaeus* females (<2 weeks old), kept without prey and plant material in a glass tube (Ø 23 mm × 120 mm), for at least 15 h, were used to assess olfactory responses to the volatiles of potted (Ø 100 mm × 200 mm) gerbera plants (cv Kimsey mini) either clean or infested with *T. vaporariorum* or *E. americanus* (ca. 200 per plant). The bioassays were carried out in a Y‐shaped Pyrex tube (internal Ø 23 mm) consisting of an entry arm, 250 mm long, and two side arms, 200 mm long (70° angle between arms), and positioned vertically as in other studies with Dicyphini (Moayeri *et al*., 2006; Ingegno *et al*., 2011). Each side arm was connected by means of silicone tubes and sealed joints to a glass cylindrical chamber (Ø 130 mm × 500 mm) as a container of the volatile source. A unidirectional airflow was generated by an air pump (Air 275R, Sera, Germany) that pushed air into the set‐up. Before reaching the chambers with the volatile sources, air passed in chronological order through a polytetrafluoroethylene membrane (HEPA filter, 0.22 μm, Ø 50 mm) to filter airborne particles, through a flow meter (EK‐2NRK, Comer, Italy) to set a constant airflow and through deionized water for humidification. Although pushing air into the set‐up can result in turbulence at the junction of the Y‐tube olfactometer, this did not impede the performance of *M. pygmaeus* in our set‐up (Ingegno *et al*., 2011). The chambers with volatiles were placed behind a black panel, preventing the predator females to see the plants during the bioassays in the Y‐tube. In all experiments, the rate through the flow meter was set at 2.5 L per minute and measured at the downwind end with a digital anemometer (TA‐410, PCE Group, Italy) to check for leaks. Before each trial, the air flow was established in the Y‐tube by adjusting the flow rate by regulating the volume of air pumped and the flow meter. Each female was observed until it had walked at least 60 mm up one of the side arms or until 10 min had elapsed; if it did not choose a side arm within 10 min, the female was considered as “no choice” and not included in the subsequent data analysis. This was the case for 1 or 2 individuals per set of 20 responding individuals. Individuals were evaluated only once to prevent any behavioral changes through experience. Each volatile source consisted of one potted gerbera plant (clean or infested), and infested plants were grown separately from noninfested plants. After five adults had made a choice, the volatile sources were switched between the left‐hand and right‐hand arms to minimize any spatial effect on choices. The Y‐tube was cleaned with mild soap and alcohol (70% v) and sterilized in an autoclave at 120 °C for 20 min. After testing 20 responses, the volatile sources were replaced with a new set of plants, so three sets of plants were tested per pair of volatile sources (3 replicates per experiment). Three experiments were done: clean gerbera versus gerbera infested with poinsettia thrips, clean gerbera versus gerbera infested with whiteflies, and gerbera infested with thrips versus gerbera infested with whiteflies. The olfactory bioassays were conducted at 25 ± 2 °C, 55% ± 2% RH, and 350 lux.

We first assessed whether there was a significant preference for one side of the olfactometer with a satiated log‐linear model for contingency tables with Generalized Linear Models (GLM) using a Poisson error distribution (log link) (Crawley, 2013) with side, replicate and their interaction as factors. We found no significant preference for one of the sides in any of the replicates. Subsequently, we tested per experiment whether the choice of the predators differed significantly among replicates, using a similar model, but with volatile source, replicate and their interaction as factors. A significant interaction between the volatile and replicate would indicate that the preference of the predators varied significantly among replicates, and a significant effect of replicate on the preference would indicate that the overall preference was more strongly determined by some replicates. This was never the case. To subsequently test whether the predators had a significant preference for one of the volatiles, models were subsequently simplified by removing the interaction between volatile and replicate. All statistical analyses were performed with R (R Development Core Team, 2017).

### Population dynamics on gerbera plants

A greenhouse trial was conducted to assess possible predator‐mediated interactions between *T. vaporariorum* and *E. americanus* on gerbera plants with the predator *M. pygmaeus* and possible resource competition on gerbera plants without predators. The experiment was carried out during summer in a greenhouse compartment of 98 m^2^ of Wageningen UR Greenhouse Horticulture. Three‐year‐old gerbera plants (cv Optima, Florist Holland B.V., de Kwakel, the Netherlands) were grown individually in 10 L pots with coco peat. Nutrients for the plants were provided through drip irrigation. To standardize plant size, we removed all flowers and old leaves so that all plants had 30 young leaves at the start of the experiment. Each gerbera plant was subsequently enclosed in a mesh cage (600 × 600 × 900 mm) made of fine gauze (mesh size 500 μm), representing an experimental unit. Plants were each infested with either 40 adults of *T. vaporariorum*, 25 adults of *E. americanus*, or a mix of both in the same densities of each pest as those used for the single pest treatment. This introduction was repeated after one week. Three pairs of 1‐week‐old adult predators per plant were released on half of the plants, once in the second and once in the third week after first pest introduction. Each experimental unit was placed within the greenhouse using a randomized complete block design with the following treatments: whiteflies only, thrips only or a mixture of both pests. These three treatments were crossed with or without the predator. These six treatments were each replicated six times; thus, 36 experimental units were used in total. Densities of whiteflies, thrips, and predators were monitored every two weeks for 10 consecutive weeks using magnifying glasses (10 ×). In the case of *T. vaporariorum*, the total numbers of larvae, pupae and adults were counted on five randomly selected leaves per experimental unit. In the case of *E. americanus* and *M. pygmaeus*, densities were assessed by counting juveniles and adults on the entire plant. During the experiment, all plants were sprayed twice with the fungicide triflumizole (Rocket®) to control a light infestation by powdery mildew. All flowers produced during the trial were removed after they had matured and carefully observed to detect any possible deformation caused by predator feeding.

Temperature and humidity in the greenhouse compartment were recorded every 5 min using a climate recorder (Hoogendoorn Growth Management, Vlaardingen, the Netherlands) throughout the experiment in a similar cage used for the experiment. The average temperature and RH during the experiment were 21.3 °C (range 15.4−36.9 °C) and 65% (range 20%–90%), respectively.

Differences in population dynamics of whiteflies, thrips, and predatory bugs among treatments were analyzed over time using a linear mixed effects model (lme of the package nlme (Pinheiro *et al*., 2014) with treatment, time and their interaction as fixed factors and replicate (plant) as random factor. Significance of interactions and factors was assessed by removing them from the model and testing with the ANOVA function of R. Contrasts among treatments were assessed with general linear hypothesis testing with the lsmeans package (Lenth, 2016). All statistical analyses were performed with R (R Development Core Team, 2017).

## Results

### Predation rates

Females of *M. pygmaeus* consumed both *T. vaporariorum* pupae and *E. americanus* adults when offered a mixture on leaf discs (one‐sample *t*‐test; *t* = 0.74, *P* = 0.48, with Manly's index [*β*] = 0.57 [± SE 0.072] for *T. vaporariorum* pupae). The mean number of consumed *T. vaporariorum* pupae and *E. americanus* adults by *M. pygmaeus* was 21.0 (±SE 2.30) and 21.6 (±SE 5.14), respectively.

### Response to plant volatiles

**Figure 1:**
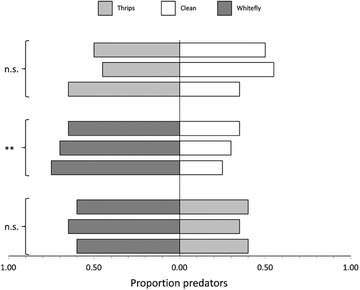
Response of females of *Macrolophus pygmaeus* in a Y‐tube olfactometer to the volatiles of potted gerbera plants (Gerbera hybrid, cv Kimsey mini). The top three bars show the results of three replicates, in each of which 20 adult females chose between the volatiles of a plant infested with thrips (light gray bars) and a clean plant (white bars). The middle three bars refer to three replicates with a choice between a plant infested with whiteflies (dark gray bars) and clean plant, and the bottom three bars are of three replicates with a choice between plants infested with thrips and plants infested with whiteflies. Each replicate was done with a new set of plants. Symbols next to the accolades denote overall significance of the preference (**P < 0.01; n.s.: not significant *P* > 0.05).

*Macrolophus pygmaeus* was not significantly attracted by volatiles from potted gerbera plants infested by thrips compared to noninfested plants (Fig. 1, top 3 bars, GLM, *χ* ^2^ = 0.27, df = 1, *P* = 0.61). In contrast, the predators were significantly attracted to plants infested by whiteflies compared to noninfested plants (Fig. 1, middle 3 bars, *χ* ^2^ = 9.87, df = 1, *P* = 0.0017). When offering gerbera plants infested by whiteflies together with gerbera plants infested by thrips, the predators had no significant preference for whitefly‐infested plants (Fig. 1, bottom 3 bars, *χ* ^2^ = 3.30, df = 1, *P* = 0.069).

### Population dynamics on gerbera plants

**Figure 2:**
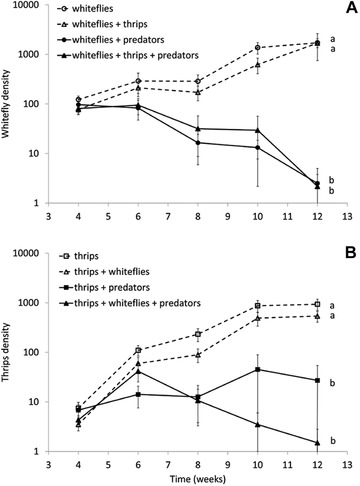
Population dynamics of *Trialeurodes vaporariorum* and *Echinothrips americanus* either alone or together on gerbera plants, and in the presence or absence of the predator *Macrolophus pygmaeus*. Shown are log(numbers) of whiteflies (A) and thrips (B) through time. Plants were infested with the pests in weeks 1 and 2; predators were released in weeks 2 and 3. Data shown are the mean (± SE) densities of (A) larvae and pupae of whiteflies on five randomly selected leaves and (B) larvae, pupae, and adults of thrips per plant. Different letters next to the curves indicate overall significant differences among treatments (contrasts after LME).

Both whitefly and thrips densities on single gerbera plants were reduced to low levels by *M. pygmaeus*, whereas densities increased to high levels without predators (Fig. 2). Whitefly densities differed significantly among treatments over time (Fig. 2A, LME, interaction of treatment with time: *χ* ^2^ = 75.5, df = 3, *P* < 0.0001). This interaction was caused by the densities of whiteflies increasing with time in the two treatments without predators and decreasing in the presence of predators. There was no significant effect of the presence of thrips on the densities of whiteflies (Fig. 2A).

Similar results were found for thrips. Thrips densities differed significantly through time among treatments (Fig. 2B, LME, interaction of treatment with time: *χ* ^2^ = 71.0, df = 3, *P* < 0.0001), caused by densities increasing in the absence of predators and remaining low in their presence. There was no significant effect of the presence of whiteflies on the densities of thrips, but there was a trend of higher thrips densities in the presence of whiteflies plus predators in the first weeks compared to the treatment with whiteflies and predators without thrips, and an opposite trend in the last weeks of the experiment (Fig. 2B). However, the difference between the thrips densities between the two treatments was not significant at any time point (contrasts after an LME with treatment and time as categorical factors and block as random factor).

**Figure 3:**
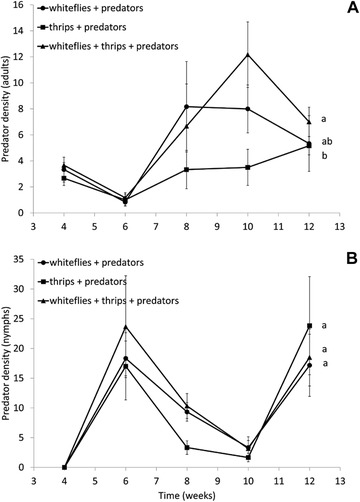
Population dynamics of the predator *Macrolophus pygmaeus* on gerbera plants with *Trialeurodes vaporariorum*, *Echinothrips americanus* or both pests. Plants were infested with the pests in weeks 1 and 2, and predators were released in weeks 2 and 3. Shown are the mean (±SE) densities of (A) adults and (B) nymphs per plant. Different letters next to the curves indicate overall significant differences among treatments (contrasts after LME).

Densities of adult *M. pygmaeus* differed significantly among treatments (LME: *χ* ^2^ = 6.57, df = 2, *P* = 0.038) (Fig. 3A) and through time (LME: *χ* ^2^ = 16.4, df = 1, *P* < 0.0001), but there was no significant interaction between these two factors (LME: *χ* ^2^ = 0.572, df = 2, *P* = 0.75). Densities were significantly lower with thrips as the only prey than with the two prey, and densities with whiteflies alone were intermediate (Fig. 3A). The number of predator nymphs did not differ significantly among treatments (LME: *χ* ^2^ = 1.25, df = 2, *P* = 0.53) (Fig. 3B), there was no significant interaction of treatments with time (LME: *χ* ^2^ = 0.133, df = 2, *P* = 0.93), but densities of nymphs varied significantly through time (*χ* ^2^ = 16.9, df = 1, *P* < 0.0001).

The gerbera plants produced on average 17–20 flowers over a period of 10 weeks during the experiment. None of the flowers showed any obvious deformation that might have arisen through plant feeding by the predators. Thrips damage to the flowers was not observed; poinsettia thrips typically feed on leaves.

## Discussion

The results of this study show that the omnivorous *M. pygmaeus* is an excellent predator of *T. vaporariorum* and *E. americanus* on single gerbera plants in cages. Both pests were controlled effectively to low levels by this predator, whereas densities reached high levels in treatments without predators. To our knowledge, this is the first study to describe effective control of *E. americanus* by a predatory mirid; earlier studies showed good control by anthocorid predatory bugs (Opit *et al*., 1997; Ramakers *et al*., 2000).

We did not find evidence for predator‐mediated interactions between *T. vaporariorum* and *E. americanus*. Based on the theory of apparent competition (Holt, 1977), lower pest densities would be expected in the long term with both pests present than with each pest separately. In theory, the opposite effect could occur in the short term, that is, higher densities when both pests are present than with each of them separately (“apparent mutualism”) (Holt, 1977; Abrams & Matsuda, 1996; van Maanen *et al*., 2012b), but there is not much evidence for this (Chaneton & Bonsall, 2000). Our results show no significant evidence for apparent competition or apparent mutualism: with or without the thrips, whiteflies were controlled very well, and there were no indications of short‐term positive effects or long term negative effects of thrips on whiteflies. In the case of *E. americanus*, there was a trend of higher thrips densities in the presence of whiteflies and the predators than without whiteflies in the first weeks, indicative for apparent mutualism, and an opposite trend in the last weeks of the experiment, suggesting apparent competition, but these differences were not significant.

The adult predator densities were significantly lower through time in the treatment with only thrips as prey than in the treatment with both pests, particularly in the period of week 8–10 (Fig. 3A), whereas the number of nymphs did not differ significantly among treatments (Fig. 3B). This could be explained by the higher initial prey densities in the treatment with both pests. The reason that we did not find strong patterns of apparent competition between the two pests might have been the plant‐feeding behavior of the predators, because it makes the predators less dependent on the presence of prey for their survival and development. Even when plants have low nutritional value, they may support predator survival and decrease juvenile developmental time (Gillespie & McGregor, 2000). Overall, the plant‐feeding behavior of *M. pygmaeus* might reduce the effects of apparent competition. However, other studies with this predator have shown clearer effects of apparent competition: whiteflies were controlled better on tomato plants by *M. pygmaeus* in the presence of the South American moth *T. absoluta* than on plants without this moth (Bompard *et al*., 2013; Jaworski *et al*., 2015). This suggests that effects of apparent competition with omnivorous predators probably also depend strongly on plant quality and possibly also on the induction of plant defences, which differs among herbivore species (Kant *et al*., 2015).

In the population experiment, both pest species were present on single plants in the treatment with both pests, whereas in reality, pests might occur on separate plants. Preference of the omnivore for plants with certain pests might then play a greater role. In the laboratory, both prey were equally preyed upon when offered together, but the olfactometer tests showed that the predators have a slight, but nonsignificant preference for plants infested by whiteflies. Thus, when pests are spatially separated in a crop, this preference might cause the temporary release of thrips from predation. However, the difference in preference for plants with either of the two pests was not significant. Hence, the lack of preference for plants with thrips relative to clean plants may not be a serious problem. Furthermore, it is possible that the predators would learn to respond to the volatiles of plants infested with thrips (Desurmont *et al*., 2018); this requires further research.

From a practical point of view, it seems recommendable to use *M. pygmaeus* for the control of *T. vaporariorum* and *E. americanus* in gerbera. On single plants, both pests were controlled well, either with or without the other pest. However, some caution is needed. Besides the possible effects of the spatial separation of the two pests in a crop outlined above, another aspect that needs further research is the fact that the plant‐feeding behavior of this predator can potentially cause some flower damage. Although we did not observe this here, it has been observed at high predator densities (Messelink & Leman, personal observations). This problem might be solved by using other species of predatory mirids that feed less on plants or flowers (Castañé *et al*., 2011). The positive effect of plant feeding on the persistence of predators might then also disappear, but this can be solved by providing alternative food sources such as *Artemia* cysts (Messelink *et al*., 2014).

In conclusion, we show that predator‐mediated interactions between whiteflies and thrips on gerbera plants are weak, and both pests are controlled well, either alone or together. The plant‐feeding behavior of their shared predator *M. pygmaeus* might have reduced the dependency of predator survival and reproduction on pests densities, thereby weakening potential predator‐mediated effects between the pests.

## Disclosure

All authors disclose no potential conflicts of interests, including specific financial interests and relationships and affiliations relevant to the subject of this manuscript.
